# The use of complementary and alternative medicine by women experiencing menopausal symptoms in Bologna

**DOI:** 10.1186/1472-6874-10-7

**Published:** 2010-02-27

**Authors:** Francesco Cardini, Grazia Lesi, Flavia Lombardo, Corinne van der Sluijs, MSCG - Menopause Survey Collaborative Group

**Affiliations:** 1 Health and Social Agency of Emilia Romagna Region, Bologna, Italy; 2 Bologna Local Health Unit, Bologna, Italy; 3 CNESPS - National Center for Epidemiology, Health Surveillance and Promotion; Italian National Health Institute, Rome, Italy; 4 CompleMED, Centre for Complementary Medicine Research, University of Western Sydney, Australia

## Abstract

**Background:**

The present study describes Complementary and Alternative Medicine (CAM) use amongst Italian women transitioning through menopause. Popularity and perceived effectiveness of CAM treatments, use of pharmaceutical medications, characteristics of CAM users, the extent of communication between medical practitioners and women about their use of CAM, and variables associated with CAM use were also investigated.

**Methods:**

Women, aged 45-65 years attending Family Planning and Women's Health clinics or Menopause Centres in Bologna were invited to complete a voluntary, anonymous, self administered questionnaire, which was used in a previous study in Sydney. The questionnaire was translated and adapted for use amongst Italian women. Data on general demographic and health characteristics, menopause related symptoms and the use of CAM and pharmaceutical treatments during the previous 12 months were collected.

**Results:**

In total, 1,203 women completed the survey, of which 1,106 were included in the final sample. Of women who had symptoms linked with menopause and/or used remedies to alleviate symptoms, 33.5% reported to have used CAM. Among these, 23.5% had consulted one or more practitioners and 24% had used at least one CAM product.

Approximately nine out of ten respondents reported medical practitioners did not seek information about their use of CAM; while one third of CAM users did not disclose the use of CAM to their physician. Nevertheless, medical practitioners were the most popular source of information. From the multivariate analysis, variables associated with CAM use were: professional employment, time since the last natural menses, use of CAM for conditions other than menopause, and presence of some severe symptoms.

**Conclusions:**

The relatively high prevalence of CAM use by women transitioning through menopause should encourage research initiatives into determining which CAM treatments are the safest and effective. The increasing and likely concomitant use of CAM with HRT and other pharmaceuticals underlines the need for the implementation of a surveillance system to report and monitor possible drug-herb adverse events. The discrepancy between women preferring to seek information about CAM from their medical doctor and the difficulties noted in communication between doctor and patient should encourage educational initiatives on CAM by health-care agencies and institutions.

## Background

A significant proportion of women experience symptoms during the peri-menopause and Hormone Replacement Therapy (HRT) is often prescribed to alleviate these symptoms [1]. However, despite the effectiveness of HRT [2], many women refuse or discontinue treatment because of side effects such as vaginal bleeding, bloating and breast tenderness or due to concerns about an increased risk of cancer or other HRT-linked conditions [3-8]. Therefore, many women are seeking safer alternative therapies to relieve symptoms and improve quality of life [9-19]. A large number of these treatments come under the broad term of Complementary and Alternative Medicine (CAM). This term refers to a wide range of diagnostic and therapeutic practices whose theoretical bases are different from those of the dominant scientific medical model. While in Italy the term generally used is "Non-conventional Medicine", this article uses the term CAM in order to be consistent with terminology used by colleagues and collaborators at CompleMED for present and future work. The terms "CAM products" and "CAM treatments" refer to preparations and treatments prescribed within their respective CAM domain, while the term "CAM practitioners" includes care-givers who may or may not be medical doctors.

In Italy, as in other developed countries, the range of CAM treatments for improving quality of life during the menopausal transition is diverse. Modalities include self-care techniques, treatments that require consultations with qualified professionals and/or the use of herbal or homeopathic products.

Although a number of studies have investigated the use of CAM during the menopausal transition [9-19], only one study was conducted in Italy [20], which investigated the use of HRT and other treatments for menopausal syndrome amongst a convenience sample of female medical doctors and the wives of doctors.

However, the findings from such a sample do not give a clear representation of the use of CAM. Therefore, the present study, although based on a convenience sample as well, aimed to obtain a more clear insight on the nature of CAM use amongst a larger sample of Italian women transitioning through menopause who were symptomatic or asymptomatic but taking treatments for menopausal related symptoms. We also investigated the popularity and perceived effectiveness of CAM treatments, the use of pharmaceutical medications, the characteristics of CAM users and the extent of communication between medical practitioners and women about their use of CAM.

## Methods

This project was approved by the Ethics Committee of the Local Health Unit of Bologna and was conducted in collaboration with CompleMED, the Centre for Complementary Medicine, University of Western Sydney. The questionnaire, originally developed, validated and used in a similar study [19], was translated into Italian (see Additional file 1). The original list of CAM products and CAM practitioners was modified to include modalities most likely to be used by Italian women. The 19 item questionnaire collected data on general demographic and health characteristics, menopause related symptoms and the use of CAM and pharmaceutical treatments during the previous 12 months. "Natural menses" was defined as a menstrual period not brought on by HRT or other medications. The menstrual status was determined from the date of the last natural menses as either having occurred more than 12 months ago, between 2 and 11 months or last month. The severity of symptoms related to menopause was rated according to a scale from 0 (no discomfort) to 6 (extreme discomfort). For clarity in interpreting the results, the severity scores were collapsed to produce three categories (0-1: none; 2-4: mild; 5-6: severe). We defined "CAM users" as women who had consulted at least one CAM practitioner (herbalist, nutritionist, naturopath, acupuncturist, traditional Chinese medical (TCM) doctor, homeopath or other practitioner) and/or used at least one CAM product (soy food or tablets, *Cimicifuga racemosa *preparations, *Angelica sinensis *preparations, phytoestrogens extracted from *Dioscorea villosa *or *Trifolium pratense*, traditional Chinese herbal formulae, homeopathic pills or other product). Women rated the effectiveness of each CAM treatment they used on a scale from 0 (no effect) to 6 (very effective). As with symptom severity, the perceived effectiveness scores for both practitioners and products were collapsed to produce three categories (0-1: not effective; 2-4: moderately effective; 5-6: very effective). Women were also asked where they had obtained information and advice regarding CAM and whether they had informed their doctor about their use of CAM.

Based on the results from previous studies [9,10], we calculated a sample size of approximately 1,200 women, which would allow an estimate of CAM use prevalence up to a 25% with a fixed precision of 2.5%.

This sample size was considered large enough to be able to make later comparisons with the sample recruited in Sydney [19]. Women, aged 45-65 years who were literate in Italian and who attended one of seven Family Planning and Women's Health (FP & WH) clinics of the Bologna Local Health Unit or two Menopause Centres (one located at a hospital and the other at a territorial center) were invited to complete the voluntary, anonymous and self administered questionnaire. Over two years the questionnaire was distributed to eligible women by staff, predominately midwives, of participating clinics and centers after information about the purpose of the study was given and informed consent was obtained. Women completed the questionnaire in the waiting room before or immediately after a consultation. Completed questionnaires were placed in a specifically designated closed container stationed in the same room. Women were excluded from the study if they had completed less than 80% of the questionnaire, were not within the designated age range, were asymptomatic but not taking specific treatments for menopausal symptoms or if the use or non-use of CAM was unclear.

Data was entered using the software package EpiInfo 2000. All statistical analyses were conducted by the CNESPS (National Centre of Epidemiology, Health Surveillance and Promotion, Italian National Health Institute, Rome) using the program STATA 8.0 (Stata Corporation, College Station, TX, USA). Data were expressed as percentages, except age which was expressed as the mean and standard deviation (SD). Differences between groups were assessed using the χ^2 ^test for categorical variables and either the Mann-Whitney test (two groups) or the Kruskal-Wallis test (three or more groups) for ordinal variables. In order to determine the characteristics associated to CAM use, we calculated prevalence ratios (PRs) with a multivariate Poisson regression using a robust error variance. All variables in the model were entered on the basis of a previous exploratory univariate analysis. Statistical significance was set at 5% (p < 0.05).

## Results

### Demographic and general health characteristics

General demographic and health characteristics for the total sample and by therapy used are presented on Table 1. In total, 1,203 women completed the survey, of which 1,106 were included in the final sample. Women were excluded if they were older or younger than the specified age range (n = 45), asymptomatic and not using a therapy specific for menopausal complaints (n = 32) or if the use or non-use of CAM was unclear (n = 27). The majority of respondents completed the questionnaire at a FP&WH clinic, while 14% were completed at Menopause Centers. No significant differences were observed between recruitment groups in regards to age, marital status, education, employment, menstrual status, prevalence of CAM use and communication with doctors. However, significantly more women attending a FP&WH clinic used CAM for other conditions unrelated to menopause. HRT was used more frequently by women from Menopause Centres. Respondents attending FP&WH clinics regarded their general health to be better and their symptoms to be less severe than recruits from Menopause Centres, although these differences were not relevant and not statistically significant. Since there were few significant differences between recruitment groups and because of an imbalance in numbers between these groups, we decided to conduct the analyses according to the type of therapy used to alleviate menopausal symptoms in the overall cohort.

**Table 1:** Demographic and health characteristics for the total sample and by therapy used

Variables	Total sample N = 1106	CAM N = 295	HRT N = 114	CAM & HRT N = 75	P*
**Age (at 2006), mean ± SD**	56.0 ± 5.3	55.8 ± 4.9	56.3 ± 5.2	55.6 ± 4.1	0.56
**Marital status, n (*%*)**					
Without partner	249 *(22.7)*	77 *(26.4)*	21 *(18.4)*	21 *(24.7)*	0.19
With partner	849 *(77.3)*	215 *(73.6)*	93 *(81.6)*	54 *(75.3)*	
**Education, n (*%*)**					
Primary school	453 *(41.0)*	*106 (36.2)*	30 *(26.3)*	25 *(33.3)*	
High school	473 *(42.9)*	*131 (44.7)*	57 *(50.0)*	33 *(44.0)*	0.41
University	178 *(16.1)*	*56 (19.1)*	27 *(23.7)*	17 *(22.7)*	
**Occupation, n (*%*)**					
Unemployed	454 *(41.8)*	113 *(39.0)*	49 *(44.1)*	22 *(30.1)*	
Non professional	157 *(14.5)*	40 *(13.8)*	6 *(5.4)*	7 *(9.6)*	0.05
Professional	475 *(43.7)*	137 *(47.2)*	56 *(50.5)*	44 *(60.3)*	
**Birth place, n (*%*)**					
Bologna city	586 *(53.5)*	150 *(51.4)*	71 *(62.3)*	43 *(57.3)*	
Bologna province	183 *(16.7)*	56 *(19.2)*	17 *(14.9)*	12 *(16.0)*	0.38
Other	327 *(29.8)*	86 *(29.4)*	26 *(22.8)*	20 *(26.7)*	
**Recruitment, n (*%*)**					
FP&WH Clinic **	950 *(85.9)*	269 *(91.2)*	84 *(73.7)*	56 *(74.7)*	
Menopause Center	156 *(14.1)*	26 *(8.8)*	30 *(26.3)*	19 *(25.3)*	< 0.001
**Last menstruation, n (*%*)**					
≥ 12 months	670 *(64.2)*	188 *(66.2)*	87 *(79.8)*	56 *(77.8)*	
2-11 months	140 *(13.4)*	41 *(14.5)*	10 *(9.2)*	12 *(16.7)*	0.012
Last month	192 *(18.4)*	43 *(15.1)*	8 *(7.3)*	1 *(1.4)*	
Surgical menopause	42 *(4.0)*	12 *(4.2)*	4 *(3.7)*	3 *(4.2)*	
**CAM for other conditions, n (%)**					
No	856 *(84.2)*	175 *(64.1)*	101 *(95.3)*	47 *(72.3)*	< 0.001
Yes	161 *(15.8)*	98 *(35.9)*	5 *(4.7)*	18 *(27.7)*	
**Use of other drugs, n (*%*)**					
No	567 *(51.3)*	156 *(52.8)*	71 *(62.3)*	41 *(*54.7)	0.23
Yes	539 *(48.7)*	139 *(47.2)*	43 *(37.7)*	34 *(*45.3)	
**Current health, n (*%*)**					
Poor (1-3)	104 *(9.5)*	37 *(12.6)*	8 *(7.0)*	4 *(5.4)*	
Good (4-5)	587 *(53.5)*	161 *(55.0)*	65 *(57.0)*	39 *(52.7)*	0.18
Excellent (6-7)	406 *(37.0)*	95 *(32.4)*	41 *(36.0)*	31 *(41.9)*	
**Past health, n (*%*)**					
Worse (1-3)	188 *(17.3)*	55 *(18.8)*	22 *(19.6)*	12 *(16.0)*	
Same (4-5)	574 *(52.7)*	162 *(55.5)*	59 *(52.7)*	38 *(50.7)*	0.74
Better (6-7)	328 *(30.1)*	75 *(25.7)*	31 *(27.7)*	25 *(33.3)*	
**Menopausal symptoms, n (%≥5)**:					
Severe hot flushes	187 *(18.6)*	74 *(26.6)*	12 *(12.0)*	25 *(34.7)*	0.01
Severe insomnia	214 *(21.3)*	78 *(29.7)*	19 *(18.5)*	17 *(24.3)*	0.08
Severe mood	132 *(*13.4	43 *(16.0)*	13 *(12.4)*	13 *(19.1)*	0.48

* significant statistical level for difference between CAM, HRT, CAM and HRT use groups

** FP&WH Clinic = Family Planning and Womens' Health Clinic

### Use of CAM and HRT for menopausal symptoms

The majority of women (56.2%) reported they had not used any treatment for menopausal complaints during the previous 12 months. Of women who had used remedies to alleviate symptoms, 10.3% had used HRT alone, 26.7% had used CAM exclusively, while 6.8% had used CAM in conjunction with HRT. Overall, 33.5% had used CAM during the twelve months before questionnaire completion. Among CAM users, one in five (20.5%) women had reported using CAM together with HRT, while 15.7% of CAM non users had used HRT (p < 0.05).

**Figure 1:**
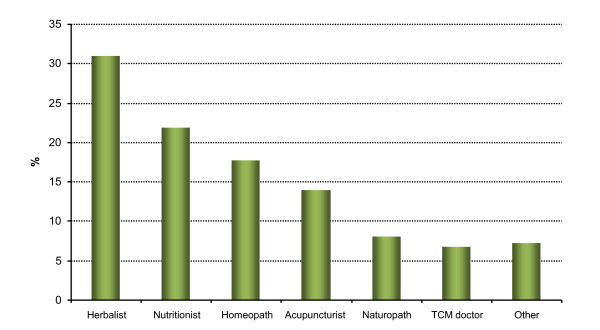
**Practitioners consulted by CAM users (n = 370)**.

**Figure 2:**
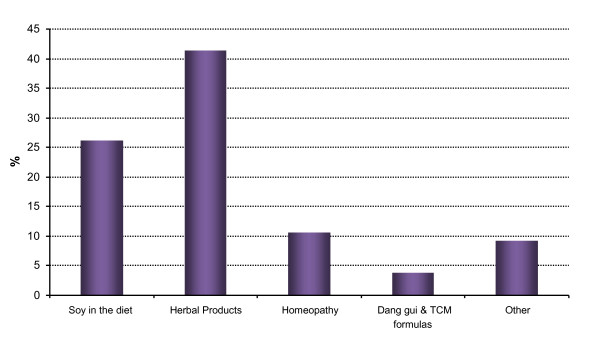
**Products used by CAM users (n = 370)**. NB: Herbal products includes *Cimicifuga *and phytoestrogens from *Dioscorea*, Soy or other plants, in pills or decoctions.

In terms of CAM practitioner use, 23.5% of women had consulted one or more practitioners; 15.5% had visited one practitioner and 8% had seen two or more. Approximately 24% of women had used at least one CAM product during the previous 12 months. Of these women, 15.8% had used one product while 7.8% had used two or more. More than half (55.5%) of the women who consulted a practitioner had also used one or more CAM products. The three most popular practitioners consulted were the herbalist, nutritionist and homeopath (Figure 1). The most popular practitioners recorded in the "other" category were the osteopath and yoga teacher. The most popular products were herbal products (which included *Cimicifuga *and phytoestrogens extracted from *Dioscorea*, soy or other plants in the form of pills or decoctions) (41.4%) and additional soy in the diet (26.2%) (Figure 2). Please note; women who took several products within a category were counted once for that category.

### Health status and perceived effectiveness of CAM modalities

The majority of women who used any therapy to alleviate menopausal symptoms reported their general health to be good. The current health status reported by women using CAM treatments did not differ when compared to women taking HRT (p = 0.14). The current perceived health of respondents not using any treatment for menopause was significantly better when compared to women who used CAM (p < 0.01). CAM users reported a number of symptoms to be more severe compared to non-users including: sleep disturbance, tension, mood, hot flushes, muscle pain, accelerated heartbeat and sweating.

**Figure 3:**
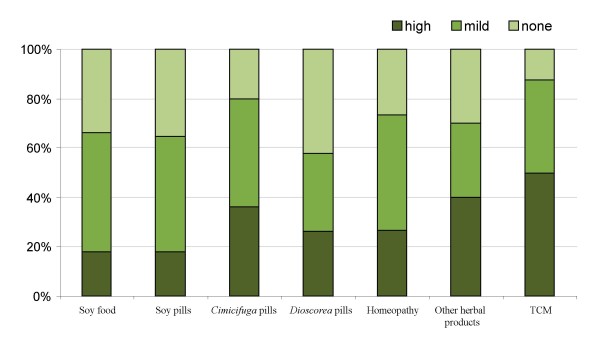
**Proportion of CAM users who found products to be highly, mildly or not effective (%.)**.

**Figure 4:**
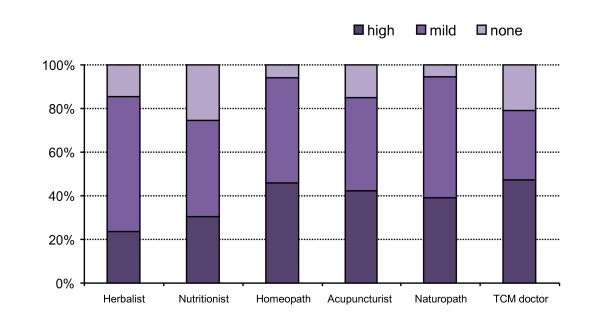
**Proportion of CAM users who found practitioners to be highly, mildly or not effective (%)**. NB: Other herbal products include mixed preparations of phytoestrogens and *Cimicifuga*.

The effectiveness of CAM products and practitioners as perceived by respondents are presented on Figures 3 and 4. The most effective products were TCM herbal formulae, *Cimicifuga *preparations and phytoestrogens. The most effective practitioners were the TCM doctor, homeopath, acupuncturist and naturopath.

### Communication about CAM with medical practitioners

Approximately nine out of ten respondents reported medical practitioners did not seek information about their use of CAM; CAM users were more often questioned by doctors than non users (19.9% and 7.9% respectively, p <0.001). Thirty one percent of CAM users did not disclose the use of CAM to their physician. It is worth noting, that 83 women (22%) who declared they had never used CAM, were in fact users as they had consulted at least one practitioner and/or used at least one product. Approximately half of the respondents (48.7%) were using prescription medications other than CAM and HRT. This proportion was similar for both CAM and non-CAM users (46.8 and 49.7% respectively).

### Sources of information about CAM

Women accessed a wide range of sources to obtain information about CAM. The most popular sources of information were medical practitioners (25%), books and herbalists (15% each), magazines (14%), friends or neighbours (13%) and pharmacists (12%). The most frequently consulted practitioner in the "other" category (completed by 8% of respondents) was the gynaecologist. Television (3%) and the Internet (2%) were the least frequently used sources of information.

### Variables associated to CAM use: multivariate analysis

Variables analyzed in a multivariate model in order to detect the characteristics associated to CAM use were: age, occupation, health status, birth place, the use of CAM for other conditions, education, date of last menses, marital status and severity of menopausal symptoms. HRT users (n = 114) and women with surgical menopause (n = 42) were excluded from the analyses. From the multivariate Poisson model, the variables associated with CAM use were determined to be: **employment**, with an increased prevalence of CAM use by 48% for women in professional employment compared to those without employment; **time since the last natural menses**, with the highest CAM prevalence amongst women whose last menses occurred between 2 and 11 months prior to the survey; **use of CAM for conditions other than menopause**, with a prevalence greater than twice amongst women who had used CAM for other conditions; and the **presence of severe symptoms**, particularly night sweats and sleep disorders. Statistically significant PRs(95% CI) are presented in Table 2.

**Table 2:** Prevalence Ratios and 95% CI for significant associations with CAM use

		PR	*P*	(95% CI)
**Occupation **(vs none):				
	Non professional	1.03	0.85	(0.74-1.45)
	Professional	1.48	0.00	(1.17-1.87)
				
**CAM for other reasons **(yes vs no)		2.19	0.00	(1.85-2.60)
				
**Last natural menses **(vs last month)				
	2-11 months	1.73	0.00	(1.27-2.36)
	≥ 12 months	1.58	0.01	(1.12-2.25)
				
**Symptoms and discomfort **(vs none)				
Night sweats	Mild	0.93	0.61	(0.69-1.24)
	Severe	1.51	0.01	(1.09-2.09)
Insomnia	Mild	1.45	0.00	(1.14-1.86)
	Severe	1.63	0.00	(1.23-2.16)

## Discussion

One third of our sample, including women aged 45-65 years and attending FP & WH Clinics or Menopause Centers in Bologna, reported to have used at least one CAM modality for menopausal complaints during the 12 months prior to the survey. Previously conducted studies investigating the use of CAM amongst menopausal women reported a wide range in prevalence rates [9-12]. These differences may be due to varying research methodologies and/or the number of modalities included as CAM. Nevertheless, when compared to the outcomes of studies incorporating similar methodologies and large sample sizes, our results indicate that CAM is utilized less frequently in Bologna [9-12,19]. According to the 2005 Multiscope Survey conducted by the Italian National Institute of Statistics (ISTAT), 20.4% of Italian women aged 45-55 years, and 17% of women aged 55-64 years used at least one CAM treatment during the previous three years [21]. The higher prevalence of use reported by our respondents may be due to different inclusion criteria, as we selected for women with menopausal symptoms. Furthermore, sociological factors may also play a role in the uptake of CAM use in Bologna. The national Multiscope Survey [21] determined that consumers of CAM tended to be middle-aged, better educated and have higher disposable incomes than non-users. CAM use was also found to be more prevalent in richer north-eastern regions of Italy [21,22]. Our survey was conducted in an affluent area with adequate medical health care services and facilities, therefore the use of CAM for menopausal complaints in Bologna may be significantly higher than other areas of Italy.

We found that respondents in Bologna were more likely to take CAM products than to see a practitioner, but this difference is less than in other surveys conducted abroad [9,19]. This could be due to the fact that in Italy CAM products are sold only in pharmacies or herbalist's shops, while in other countries these products are also sold in supermarkets.

Respondents preferred taking soy as food and/or phytoestrogens rather than other CAM products. The addition of soy containing foods to the diet is an easy and inexpensive way to increase phytoestrogen consumption [9,19]. Therefore, our observation supports the hypothesis that CAM users orient their choices towards simple and less expensive products. Respondents regarded traditional Chinese herbal products, phytoestrogens and *Cimicifuga *to be the most effective products. Despite individual clinical trial outcomes suggesting that phytoestrogens isolated from soy and other herbs may confer a moderate effect in alleviating menopausal symptoms, the effectiveness of isoflavones and phytoestrogens were not assessed and therefore remains controversial [23,24]. Contrary to the findings of the Multiscope Italian survey (which refers to the general population), our respondents consulted with herbalists and nutritionists, more often than homeopaths and manipulative therapists such as osteopaths, chiropractors and massage therapists [21,22]. The findings that manipulative treatments were not commonly utilised for menopausal complaints by respondents contrasts those of the original survey conducted in Sydney [19].

Our results indicate that, amongst both CAM users and non users, almost one in two respondents used pharmaceutical medications within the past year. Approximately 20% of CAM users had taken HRT during the previous 12 months. However, we were unable to ascertain if pharmaceuticals were used concurrently or sequentially with CAM treatments. Nevertheless, these significantly high usage rates indicate a need to improve our knowledge of and to strengthen our surveillance measures for detecting possible interactions between CAM and pharmaceutical medications [25,26]. Our results confirm a lack of communication between physicians and patients on the use of CAM [27], which makes monitoring the use of medications difficult. This lack of communication was bi-directional, as 9 out of 10 respondents indicated that GPs did not discuss their use of CAM, while two out of five CAM users disclosed their use of alternative therapies to their doctor. It was interesting to note the high number of missing (8%) and inconsistent answers (22%) to the question "do you usually tell your medical doctor when using CAM?". The fact that one in five CAM users answered "I have never used CAM" indicates a gap in communication which may be due to either a confusion over the definition of "complementary medicine" or the purposeful concealment of the use of CAM, possibly due to the belief that declaring the use of CAM may negatively interfere with the healing relationship between doctor and patient. This worry is rather common and has been described in previous studies. A national survey conducted in the U.S. [28] found more than one third of respondents declared they did not disclose the use of CAM to their doctors because "the doctor would not understand" (20%), "would disapprove of or discourage CAM use" (14%) or "might not continue as their provider" (2%). Despite these concerns, participants in our study indicated the medical doctor as the most frequently consulted source of information about CAM. The Internet was one of the least consulted sources, possibly due to the age of respondents, who are more likely to be computer illiterate. Use of the Internet as a source of information may increase in popularity as successive more computer-literate generations reach menopausal age. Since Italian television has dedicated little time to the exploration of CAM, television as a source of information was rated poorly.

The most significant variables associated with CAM use were: having used CAM for other conditions, the presence of severe symptoms, being post menopausal and professionally employed. If a woman had used CAM for other conditions, she was around two times more likely to use CAM for menopausal complaints: this may indicate a level of satisfaction with previous CAM use, warranting its use for menopausal symptoms. Postmenopausal women with severe symptoms were more likely to be users of CAM. The disruption of sleep due to night sweats (more than any other symptom, including hot flushes) may instigate the use of alternative treatments, as women may no longer tolerate symptoms that affect daytime mood and performance. Finally, women who have higher disposable incomes were more likely to use CAM, which is consistent with the findings of previous surveys [21].

### Strengths and limitations

To our knowledge, this is the first comprehensive survey conducted in Italy that explores the use of CAM by symptomatic women or by women who were asymptomatic but taking menopause specific treatments during the menopausal transition. The Italian translation of the questionnaire was validated for face and content by a panel of experts who reviewed and adapted it to the Italian context; it was not validated for repeatability and reliability, given that this had already been done in Australia for the original questionnaire (19). We acknowledge that some limitations of our study make the extrapolation of our results to the general population difficult. The use of convenience sampling of women from a number of health clinics may have over inflated the use of CAM and the reporting of menopausal symptoms. Furthermore, the voluntary self administration of the questionnaire may have selected for respondents who were more interested in CAM thereby overestimating the true prevalence of CAM use. Despite these limitations, we believe our study is valuable in highlighting the popularity of CAM use by symptomatic menopausal women. Strengths of the survey included the large sample size of women recruited throughout the Bologna region, and that we explored the use and perceived effectiveness of both CAM practitioners and products.

## Conclusions

The results of this survey highlight a number of issues relating to the use of CAM during the menopausal transition for women in Bologna. The increasing and likely concomitant use of CAM with HRT and other pharmaceuticals may necessitate a need for the implementation of a surveillance system to report and monitor possible drug-herb adverse events.

The discrepancy between women preferring to seek information about CAM from their medical doctor and the difficulties noted in communication between doctor and patient should encourage educational initiatives on CAM by health-care agencies and institutions. Patients should be informed about the risks and benefits of CAM, while health care professionals (particularly primary care practitioners) should be encouraged to communicate effectively with patients about their use of CAM and pharmaceuticals, and clearly record all medications consumed, signaling any possible adverse events or interactions.

The high prevalence of CAM use by women transitioning through menopause should encourage research initiatives into determining which CAM treatments are the safest and effective, giving priority to treatments identified by the literature as the most promising and the most popular; however keeping in mind that popular treatments may not always equate to the most effective.

## Competing interests

The authors declare that they have no competing interests.

## Authors' contributions

FC conceived of the study, and participated in its design and coordination and drafted and wrote the manuscript. GL organized and supervised the distribution and collection of the questionnaires, and the data input. FL performed the statistical analysis. CvdS designed and realized the original study (conducted in Sydney), authorized the adaptation and the use of the questionnaire, participated in the design of this study, and revised the manuscript. All members of the Menopause Survey Collaborating Group (MSCG) contributed in different ways to the study. All authors read and approved the final manuscript.

## Pre-publication history

The pre-publication history for this paper can be accessed here:

http://www.biomedcentral.com/1472-6874/10/7/prepub

## Supplementary Material

Additional file 1 **CAM - Menopause questionnaire (Italian).pdf**. questionnaire administered in the study. Click here for file
