# Supplementary material for: The use of complementary and alternative medicine by women experiencing menopausal symptoms in Bologna
Source: BMC Womens Health. 2010 Feb 27;10:7. doi: 10.1186/1472-6874-10-7 (PMC2846842; doi:10.1186/1472-6874-10-7)
Supplement: Additional file 1 — CAM - Menopause questionnaire (Italian).pdf. questionnaire administered in the study. [file 1472-6874-10-7-S1.PDF]

SERVIZIO SANITARIO REGIONALE
EMILIA-ROMAGNA
Azienda USL di Bologna

Questa ricerca è eseguita
in collaborazione con:

University of Western Sydney

CompleMED Research Centre

La salute della donna

tra i 45 e i 65 anni.

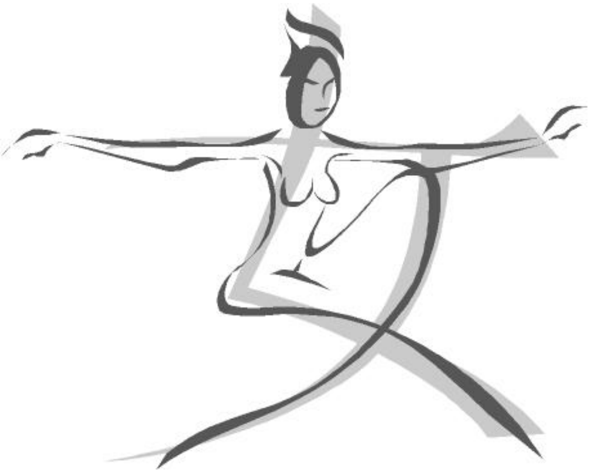

Se siete donne di un'età compresa tra i 45 e i 65 anni, questo questionario fa per voi! Esso mira a scoprire quanto siano diffusi i trattamenti complementari per il controllo dei sintomi della menopausa e a comprendere quanto le donne li considerano efficaci. I trattamenti complementari (detti anche non convenzionali) sono quelli non ormonali e non farmacologici, e comprendono quelli a base di erbe medicinali, la naturopatia, l'omeopatia, l'agopuntura, l'alimentazione, la chiroterapia, l'osteopatia, il massaggio, lo yoga, la meditazione, e molti altri meno diffusi di quelli elencati.

Vi chiediamo di dedicare **10 - 15 minuti** del vostro tempo per rispondere al questionario.

Il questionario è suddiviso in 3 parti:

- La vostra salute
- I trattamenti utilizzati durante la menopausa
- Notizie riguardo alla vostra persona (istruzione, professione etc.)

Le risposte al questionario sono **volontarie e anonime**, quindi **non scrivete il vostro nome** in nessuna parte del questionario.

Grazie della collaborazione!

Ai sensi del Testo Unico in materia di protezione dei dati personali di cui al D.Lgs. 196/2003 ("Codice sulla Privacy"), segnaliamo che i dati vengono raccolti esclusivamente per gli scopi scientifici indicati nel questionario stesso.

Parte 1: La vostra salute

1. In generale, come valutate la vostra salute in questo momento?
(Barrate solo una casella)

Scarsa ←→ **Ottima**

1 ☐ 2 ☐ 3 ☐ 4 ☐ 5 ☐ 6 ☐ 7 ☐

2. Rispetto ad un anno fa, come valutate la vostra salute in questo momento?
(Barrate solo una casella)

Peggiore ←→ **Migliore**

1 ☐ 2 ☐ 3 ☐ 4 ☐ 5 ☐ 6 ☐ 7 ☐

3. State assumendo **medicinali** prescritti dal vostro medico?
(Barrate solo una casella)

☐ Sì →

☐ No

Se sì, indicate di seguito il medicinale che state assumendo.

____

____

4. Negli ultimi 12 mesi vi stete sottoposte alla Terapia Sostitutiva Ormonale (TSO) per ridurre i sintomi della menopausa?
(Barrate solo una casella)

☐ Sì

☐ No

☐ Mi sottopongo alla TSO per altri motivi

5. Quando si è verificato il vostro ultimo ciclo mestruale **naturale**?
Un ciclo mestruale naturale è un ciclo mestruale **non** causato dalla Terapia Sostitutiva Ormonale o da altri trattamenti.
(Barrate solo una casella)

☐ 12 mesi fa o più

☐ da 2 a 11 mesi fa

☐ il mese scorso

☐ ho subito l'asportazione chirurgica dell'utero all'età di .... anni

6. Negli ultimi 12 mesi in quale misura vi hanno procurato fastidio i sintomi seguenti? Utilizzate la scaletta sottostante per valutare i sintomi accusati.

   Completare per ogni sintomo.

	Nessun fastido ←						→ Molto fastidio
(Barrate solo una casella per ogni sintomo)	**0**	**1**	**2**	**3**	**4**	**5**	**6**
Vampate di calore	☐	☐	☐	☐	☐	☐	☐
Sudorazioni notturne	☐	☐	☐	☐	☐	☐	☐
Battito cardiaco accellerato o più intenso	☐	☐	☐	☐	☐	☐	☐
Sensazione di tensione **o** nervosismo	☐	☐	☐	☐	☐	☐	☐
Difficoltà del sonno	☐	☐	☐	☐	☐	☐	☐
Attacchi di panico	☐	☐	☐	☐	☐	☐	☐
Cambiamenti di umore	☐	☐	☐	☐	☐	☐	☐
Sensazione di vertigini **o** debolezza	☐	☐	☐	☐	☐	☐	☐
Cefalea	☐	☐	☐	☐	☐	☐	☐
Dolore muscolare e alle articolazioni	☐	☐	☐	☐	☐	☐	☐
Formicolii	☐	☐	☐	☐	☐	☐	☐
Difficoltà di respirazione	☐	☐	☐	☐	☐	☐	☐
Mestruazioni irregolari	☐	☐	☐	☐	☐	☐	☐
Infezioni vescicali	☐	☐	☐	☐	☐	☐	☐
Secchezza vaginale	☐	☐	☐	☐	☐	☐	☐
Altro ____________________	☐	☐	☐	☐	☐	☐	☐
Altro ____________________	☐	☐	☐	☐	☐	☐	☐

Parte 2: L’uso dei trattamenti complementari durante la menopausa

I trattamenti complementari (detti anche non convenzionali) sono quelli non ormonali e non farmacologici, e comprendono quelli a base di erbe medicinali, la naturopatia, l’omeopatia, l’agopuntura, l’alimentazione, la chiroterapia, l’osteopatia, il massaggio, lo yoga, la meditazione, e molti altri meno diffusi di quelli qui elencati.

7. Negli ultimi 12 mesi avete consultato qualcuno degli esperti in medicina complementare elencati qui sotto, per curare i sintomi della menopausa? Non preoccupatevi se non conoscete qualcuna delle medicine complementari elencate.

   Se **non** avete mai consultato uno degli specialisti segnate “**No**”.

(Per ciascuna persona elencata sotto barrate Sì o No)		**Se avete segnato Sì,** quanto, secondo voi, è stata efficace la cura? Per nulla efficace ←→ Molto efficace						
		**0**	**1**	**2**	**3**	**4**	**5**	**6**
Erborista o fitoterapeuta	☐ Sì<br>☐ No	☐	☐	☐	☐	☐	☐	☐
Nutrizionista o dietologo	☐ Sì<br>☐ No	☐	☐	☐	☐	☐	☐	☐
Naturopata	☐ Sì<br>☐ No	☐	☐	☐	☐	☐	☐	☐
Agopuntore	☐ Sì<br>☐ No	☐	☐	☐	☐	☐	☐	☐
Medico esperto in Medicina Tradizionale Cinese	☐ Sì<br>☐ No	☐	☐	☐	☐	☐	☐	☐
Omeopata	☐ Sì<br>☐ No	☐	☐	☐	☐	☐	☐	☐
Altro professionista consultato *(scrivere sotto)*<br>______________	☐ Sì<br>☐ No	☐	☐	☐	☐	☐	☐	☐
Altro professionista consultato *(scrivere sotto)*<br>______________	☐ Sì<br>☐ no	☐	☐	☐	☐	☐	☐	☐

8. Negli ultimi 12 mesi avete utilizzato qualcuno dei prodotti elencati qui sotto per curare o prevenire i sintomi della menopausa?
Completate prima la colonna a sinistra, poi la tabella a destra.

**Per ciascun prodotto barrate Sì oppure No**		**Se avete barrato Sì**, in quale misura il prodotto è stato efficace? Per nulla efficace ↔ Molto efficace						
		**0**	**1**	**2**	**3**	**4**	**5**	**6**
Più prodotti a base di soia nella dieta (es. latte di soia o tofu)	☐ Sì ☐ No	☐	☐	☐	☐	☐	☐	☐
Pillole a base di soia (es. Estromineral, Evestrel, Rinnova, Serena, Phytosoya, Fitogen….)	☐ Sì ☐ No	☐	☐	☐	☐	☐	☐	☐
Pillole a base di Cimicifuga Racemosa (es. Remifemin, Menovamp, Fitofem)	☐ Sì ☐ No	☐	☐	☐	☐	☐	☐	☐
Pillole a base di Dioscorrea vilosa, trifolium pratense (es Pausa)	☐ Sì ☐ No	☐	☐	☐	☐	☐	☐	☐
Prodotti omeopatici (Es. Klimactoplant)	☐ Sì ☐ No	☐	☐	☐	☐	☐	☐	☐
Cimicifuga Racemosa in decotto	☐ Sì ☐ No	☐	☐	☐	☐	☐	☐	☐
Fitoestrogeni della soia + cimicifuga racemosa (es.Climil, Fitodonna)	☐ Sì ☐ No	☐	☐	☐	☐	☐	☐	☐
Pillole o decotti a base di Dang gui (Angelica sinensis)	☐ Sì ☐ No	☐	☐	☐	☐	☐	☐	☐
Prodotti della Medicina tradizionale cinese: (es Liu Wei Pils etc.)	☐ Sì ☐ No	☐	☐	☐	☐	☐	☐	☐
Altri prodotti utilizzati (Scrivere sotto) ______________	☐ Sì	☐	☐	☐	☐	☐	☐	☐
Altri prodotti utilizzati (Scrivere sotto) ______________	☐ Sì	☐	☐	☐	☐	☐	☐	☐

Nota bene: per nessuno dei prodotti elencati nella tabella esistono prove certe e definitive di efficacia.

9. **In questo periodo** state utilizzando la medicina complementare per curare altri problemi di salute? (La medicina complementare comprende i trattamenti menzionati nelle domande 7 e 8). *(Barrate solo una casella)*

☐ Sì

☐ No

10. Il vostro medico di base solitamente vi chiede se utilizzate la medicina complementare? *(Barrate solo una casella)*

☐ Sì

☐ No

11. Solitamente riferite al vostro medico di base quando utiilizzate la medicina complementare? *(Barrate solo una casella)*

☐ Sì

☐ No

☐ Non ho mai utilizzato la medicina complementare

12. Chi vi ha suggerito l'utilizzo della medicina complementare? *(Potete barrare più di una casella)*

☐ Non ho mai utilizzato la medicina complementare[1]
☐ Libri di medicina complementare o alternativa[2]
☐ Quotidiani[3]
☐ Riviste[4]
☐ Televisione[5]
☐ Siti internet[6]
☐ Chiroterapeuta, osteopata[7]
☐ Specialista in medicina cinese (Agopuntura e/o Fitoterapia)[8]
☐ Erborista[9]
☐ Naturopata[10]
☐ Commesso di negozio di alimentazione naturale[11]
☐ Amico o vicino di casa[12]
☐ Coniuge o parente[13]
☐ Medico [14]
☐ Farmacista[15]
☐ Altro________________________ *(scrivere a fianco)*

Parte 3: Notizie riguardanti la vostra persona

13. Qual' è la vostra data di nascita?

Giorno Mese Anno

☐☐ ☐☐ ☐☐

14. Qual' e' la vostra formazione scolastica?
(Barrate solo una casella)

- ☐ licenza elementare[1]
- ☐ licenza di scuola media inferiore[2]
- ☐ diploma di scuola media superiore[3]
- ☐ diploma professionale[4] (es: infermiere prof, ostetrica etc)
- ☐ laurea[5]

15. Quale dei seguenti gruppi descrive meglio il vostro attuale lavoro?
(Barrate solo una casella

- ☐ Personale non qualificato[1]
(esempi: addetto pulizie, portalettere, aiuto-cuoco, etc)
- ☐ Personale addetto alla vendita[2]
Es: cassiera, commessa, rapprensentante
- ☐ Commerciante o artigiano o tecnico specializzato, agricoltore[3]
Es: tipografa, fornaia etc.
- ☐ Impiegata o segretaria[4]
Es: impiegata di banca, operatrice informatica, etc.
- ☐ Professioni intellettuali specializzate[5]
Es: insegnante, contabile, assistente sociale, infermiera, programmatrice di computer
- ☐ Dirigente, manager, imprenditrice, libera professionista[6]
- ☐ Casalinga[7]
- ☐ Disoccupata o in cerca di impiego[8]
- ☐ Condizione non professionale[9] (Es: studente, pensionata)

16. Qual' è il codice di avviamento postale della località in cui vivete abitualmente?

☐ ☐ ☐ ☐ ☐

17. Qual'è il vostro stato civile attuale?
(Barrate solo una casella)

☐ Sposata[1]
☐ Convivente[2]
☐ Separata[3]
☐ Divorziata[4]
☐ Vedova[5]
☐ Nubile[6]

18. Qual è la vostra provenienza?
(barrate solo una casella)

☐ Bologna[1]
☐ Provincia di Bologna[2]
☐ Regione Emilia Romagna[3]
☐ Altre regioni italiane[4]
☐ Europa Occidentale[5]
☐ Europa dell' Est[6]
☐ Africa[7]
☐ Estremo oriente[8]
☐ Altri paesi[9] ____________________ *(scrivere a fianco)*

19. Quale lingua parlate **abitualmente** a casa?

____

Controllate di avere risposto a tutte le domande.

Inserite il questionario in una busta, sigillatela e consegnatela al responsabile della ricerca (*)
Vi ringraziamo per avere risposto al questionario.

Se desiderate esprimere commenti riguardo al presente questionario, scriveteli nello spazio sottostante.

____

____

____

____

____

This document was created with Win2PDF available at http://www.win2pdf.com.
The unregistered version of Win2PDF is for evaluation or non-commercial use only.
This page will not be added after purchasing Win2PDF.
